# Topically delivered 22 nt siRNAs enhance RNAi silencing of endogenous genes in two species

**DOI:** 10.1007/s00425-021-03708-y

**Published:** 2021-08-26

**Authors:** Bill Hendrix, Wei Zheng, Matthew J. Bauer, Ericka R. Havecker, Jennifer T. Mai, Paul H. Hoffer, Rick A. Sanders, Brian D. Eads, Amy Caruano-Yzermans, Danielle N. Taylor, Chelly Hresko, Janette Oakes, Alberto B. Iandolino, Michael J. Bennett, Jill Deikman

**Affiliations:** 1 Bayer Crop Science, 37437 State Highway 16, Woodland, CA 95695 USA; 2 Bayer Crop Science, 700 Chesterfield Parkway West, Chesterfield, MO 63017 USA; 3 Present Address: Bayer U.S. LLC, Research and Development, Crop Science, Biologics Pest Control, 890 Embarcadero Drive, West Sacramento, CA 95605 USA; 4 Present Address: California Governor’s Office of Emergency Services, 3650 Schriever Avenue, Mather, CA 95655 USA

**Keywords:** RNA interference, Transitive small RNAs, Systemic silencing, *Nicotiana benthamiana*, *Amaranthus cruentus*

## Abstract

**Main conclusion:**

22 nt siRNAs applied to leaves induce production of transitive sRNAs for targeted genes and can enhance local silencing. Systemic silencing was only observed for a *GFP* transgene.

**Abstract:**

RNA interference (RNAi) is a gene silencing mechanism important in regulating gene expression during plant development, response to the environment and defense. Better understanding of the molecular mechanisms of this pathway may lead to future strategies to improve crop traits of value. An abrasion method to deliver siRNAs into leaf cells of intact plants was used to investigate the activities of 21 and 22 nt siRNAs in silencing genes in *Nicotiana benthamiana* and *Amaranthus cruentus*. We confirmed that both 21 and 22 nt siRNAs were able to silence a green fluorescent protein (*GFP*) transgene in treated leaves of *N. benthamiana*, but systemic silencing of *GFP* occurred only when the guide strand contained 22 nt. Silencing in the treated leaves of *N. benthamiana* was demonstrated for three endogenous genes: *magnesium cheletase subunit I* (*CHL-I*), *magnesium cheletase subunit H* (*CHL-H*), and *GENOMES UNCOUPLED4 (GUN4)*. However, systemic silencing of these endogenous genes was not observed. Very high levels of transitive siRNAs were produced for *GFP* in response to treatment with 22 nt siRNAs but only low levels were produced in response to a 21 nt siRNA. The endogenous genes tested also produced transitive siRNAs in response to 22 nt siRNAs. 22 nt siRNAs produced greater local silencing phenotypes than 21 nt siRNAs for three of the genes. These special properties of 22 nt siRNAs were also observed for the *CHL-H* gene in *A. cruentus*. These experiments suggest a functional role for transitive siRNAs in amplifying the RNAi response.

**Supplementary Information:**

The online version contains supplementary material available at 10.1007/s00425-021-03708-y.

## Introduction

RNA interference (RNAi) is a gene silencing mechanism used by plants and other eukaryotes to regulate the expression of endogenous genes, to silence transposons (and other heterochromatic regions), and defend against virus infection (Baulcombe 2004). It is achieved by the action of a small RNA (sRNA) with complementarity to the target gene sequence bound to an argonaute (AGO) protein to form an RNA-induced silencing complex (RISC). Silencing can occur at the transcriptional or post-transcriptional level, depending on the specific forms of these components. RNAi has been used to create a number of valuable traits in plants, and there is great potential for future applications to crop improvement (Kamthan et al. 2015; Mezzetti et al. 2020). Recently, plants have been shown to respond to RNA applied topically to leaf surfaces (Dalakouras et al. 2016, 2020; Dubrovina et al. 2019; Demirer et al. 2020; Das and Sherif 2020; Schwartz et al. 2020), and this delivery route could expand the opportunities for using RNAi for agronomic benefit, with further optimization. In addition, topical delivery of sRNAs to plant cells provides a tool that can be used to investigate details of the RNAi mechanisms in plants.

The 21–24 nt double-stranded duplexes that initiate RNA silencing in plants are produced by Dicer-like (DCL) RNAse III family enzymes acting on various precursors, including miRNA gene hairpin-structured transcripts (Yu et al. 2017) and long dsRNAs of various sources including viruses, transposons or transgenic hairpins (Axtell 2013). These sRNA duplexes contain 2 nt 3′ overhangs (Axtell 2013). In forming the RISC, the guide strand complementary to the target mRNA is selectively bound to an AGO protein, while the passenger strand is degraded (Fang and Qi 2016). For post-transcriptional silencing, the RISC interacts with the complementary mRNA and either cleaves (slices) the complementary mRNA (Arribas-Hernández et al. 2016) or acts to repress translation (Li et al. 2013; Fang and Qi 2016).

Secondary siRNAs (also referred to as transitive siRNAs) can be produced from transcripts targeted by miRNAs or siRNAs (Moissiard et al. 2007). These secondary siRNAs may amplify silencing of the primary target or act in trans to silence members of a gene family, such as the well-studied example of NBS-LRR disease-resistance mRNAs (Axtell 2013). Secondary siRNA production requires RNA-dependent RNA polymerase 6 (RDR6) and DCL4 and is sometimes phased (Axtell 2013). While there are multiple factors that can initiate secondary siRNA production (Axtell 2013), they are produced in response to 22 nt miRNAs, while 21 nt miRNAs do not usually have this effect (Chen et al. 2010; Cuperus et al. 2010). The functional significance of 22 nt miRNAs was shown using artificial miRNAs (amiRNAs) in transgenic Arabidopsis plants, where whole-plant silencing of a targeted *chalcone synthase (CHS)* gene was only achieved with a 22 nt amiRNA, and not with the corresponding 21 nt amiRNA (McHale et al. 2013). This effect was dependent on RDR6, and the generation of phasiRNAs was demonstrated. The authors hypothesized that the enhanced silencing could be due to greater mobility of siRNAs (Tretter et al. 2008; Felippes et al. 2011) or to production of a significant amount of additional siRNAs homologous to *CHS* allowing gene knock-down throughout the plant.

There are several pathways for the generation of 22 nt siRNAs or miRNAs in plant cells. DCL2 processes 22 nt siRNAs from long dsRNAs, and its activity is critical for both the production of secondary siRNAs and the transitive silencing of transgenes (Mlotshwa et al. 2008; Parent et al. 2014; Taochy et al. 2017; Chen et al. 2018). Alternatively, 22 nt miRNAs can be generated by processing by DCL1 of an asymmetric precursor miRNA to generate a 21/22 nt miRNA (Manavella et al. 2012), by imprecise cutting by DCL1 (Li et al. 2016), or by mono-uridylation by HEN1 Supressor1 (HESO1) of a 21 nt miRNA (Fei et al. 2018). The exact structure of a miRNA or siRNA that can promote production of secondary siRNAs is unclear, and there are instances where 21 nt miRNAs can trigger production of secondary siRNAs (Manavella et al. 2012). Additionally, examples were shown where secondary siRNAs can be produced when the passenger strand of the miRNA duplex is 22 nt but the miRNA is 21 nt (Manavella et al. 2012). These researchers proposed that the critical structural feature for induction of secondary siRNAs is not the specific size of the miRNA, but asymmetry in the miRNA duplex structure. Recent work showing formation of a 22 nt miRNA that is associated with secondary siRNA production by mono-uridylation of a 21 nt miRNA (Fei et al. 2018) would support the importance of miRNA length alone, although it does not rule out other mechanisms.

Silencing by RNAi can spread from a site of initiation to distal parts of a plant. This phenomenon has been observed in multiple species using grafting of genotypes containing silencing transgenes, by initiation of local silencing using *Agrobacterium* infiltration of hairpin constructs, by expression of hairpins or amiRNAs under control of tissue specific promoters, or by topical introduction of a dsRNA (Palauqui et al. 1997; Voinnet and Baulcombe 1997; McHale et al. 2013; Dalakouras et al. 2016; Hendrix et al. 2021). The signal for systemic silencing has not been conclusively determined but is generally thought to be an RNA (Melnyk et al. 2011; Taochy et al. 2017; Zhang et al. 2019). sRNAs can move from cell-to-cell in plants through plasmodesmata or longer distances through vascular tissues (Melnyk et al. 2011; Liu and Chen 2018). Cell-to-cell movement combined with template-dependent signal amplification is another mechanism described for long-distance sRNA movement (Liang et al. 2012). Recent work has highlighted the critical role of *DCL2*, and thus 22 nt siRNAs, in systemic RNAi (Taochy et al. 2017; Chen et al. 2018). Systemic silencing of the *GFP* transgene was shown to be associated with high levels of the target gene expression (Hendrix et al. 2021).

To better understand the properties of siRNAs that can produce silencing, we used an abrasion-based delivery method to introduce dsRNAs into leaf cells of intact plants for two plant species. We confirmed that while both 22 nt siRNAs and 21 nt siRNAs produced local silencing phenotypes for a GPF transgene in *N. benthamiana* with this delivery method, only 22 nt siRNAs induced systemic silencing, while 21 nt siRNAs did not. Local silencing was achieved with both 21 and 22 nt siRNAs for 3 endogenous genes, but systemic silencing was not observed for any of the 3 endogenous genes targeted. We showed that 22 nt siRNAs produced greater local phenotypes than 21 nt siRNAs for 3 of the 4 gene targets tested. 22 nt siRNAs induced secondary sRNA production for all target mRNAs, but to different levels. Very high levels of secondary siRNAs were produced for the *magnesium cheletase H* (*CHL-H*) gene, but systemic silencing was still not observed. The low-tech abrasion-based RNA delivery method is simple to use and has utility for future study of factors required for efficient RNAi in plants.

## Results

### *GFP* transgene silencing in *N. benthamiana* by topically applied siRNA

The *GFP* transgene was silenced using a sandpaper abrasion method (Huang et al. 2018) to introduce dsRNAs into leaf cells of *N. benthamiana* line 16C (Ruiz et al. 1998). An efficacious dsRNA to silence *GFP* was selected by calculating a Reynolds score (Reynolds et al. 2004) for all possible 19 mers, and then testing high scoring sequences as 22 nt siRNAs with 2 nt 3′ overhangs in protoplasts prepared from tobacco BY-2 cells expressing a *GFP* transgene (Yang et al. 2021). The 22 nt siRNA showing the greatest silencing in protoplasts was used as the base sequence for studies using topical dsRNA treatment of intact plants (Table 1).

**Table 1:** siRNAs targeting *GFP* or control sequences

Duplexes	Sense (passenger) strand (5′–3′):antisense (guide) strand (5′–3′)	Figure/Table
Controls		
*A. palmeri EPSPS*	AUGCCAGAUGUUGCUAUGACUCUU:AAGAGUCAUAGCAACAUCUGGCAU	Figures 1, 2, 3
Scrambled	GCCGAUCAACCUCAAACUUAAU:GAAGUUUGAGGUUGAUCGGCUU	Figure 6
*N. benthamiana 16C-GFP*		
*GFP*_21/22	GGC-UCAAAGCCAACUUCAAAA:UUGAAGUUGGCUUUGAUGCCGU	Figures 1, 2, 3, 4, 5; Table 3
* GFP*_20/20	CAUCAAAGCCAACUUCAAAA:UUGAAGUUGGCUUUGAUGCC	Figure 2
* GFP*_21/21	GCAUCAAAGCCAACUUCAAAA:UUGAAGUUGGCUUUGAUGCCG	Figures 2, 4; Table 3
* GFP*_22/22	GGCAUCAAAGCCAACUUCAAAA:UUGAAGUUGGCUUUGAUGCCGU	Figures 2, 3, 4; Table 3
* GFP*_22/21	GCACUCAAAGCCAACUUCAAAA:UUGAAGUUGGCUUUGA-UGCCG	Figures 2, 4; Table 3
* GFP*_21/22_mut 9–11	GGC-UCAAA**CGG**AACUUCAAAA:UUGAAGUU**CCG**UUUGAUGCCGU	Figure 3
* GFP*_21/22_mut 10–12	GGC-UCAA**UCG**CAACUUCAAAA:UUGAAGUUG**CGA**UUGAUGCCGU	Figure 3
* GFP*_21/21_v2	GCAUCAAAGCCAACUUCAAGA:UUGAAGUUGGCUUUGAUGCCG	Figure 5

Letters in bold indicate introduced mutations

**Fig. 1:**
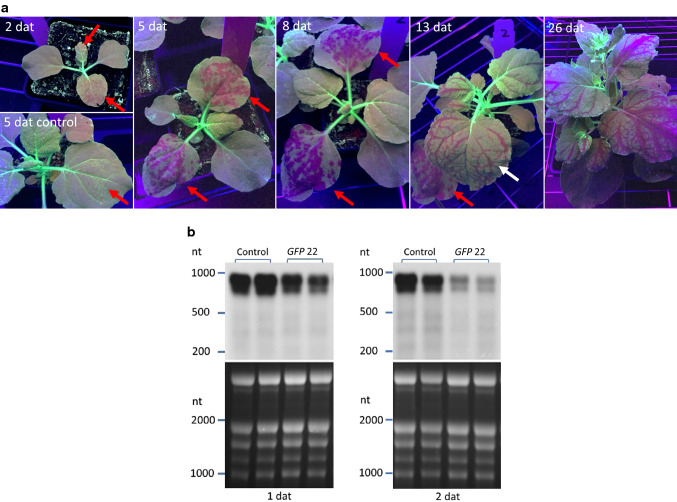
Development of *GFP* silencing over time in *N. benthamiana* plants in response to topically applied siRNAs. **a** *GFP* silencing appeared as red spots on treated leaves (red arrow) when plants were viewed under UV light as soon as 2 days after treatment (dat) and systemic silencing was visible in veins of upper leaves (white arrow) by 13 days after treatment. dsRNA was applied onto leaves 3 and 4 of 14-day old plants followed by abrasion with #600 grit sandpaper (red arrows). Plants were treated with a *GFP* 21/22 siRNA, except for the control, which was treated with a 24 nt dsRNA from the *5-enolpyruvylshikimate-3-phosphate synthase* (*EPSPS*) coding sequence from *Amaranthus palmeri*. At the indicated times after treatment, plants were photographed under UV light. **b** RNA gel blot probed with *GFP* sequence (upper panels) and gel stained with ethidium bromide (lower panels). Samples from two treated leaves were collected 1 and 2 days after treatment

GFP fluoresces green under UV light, and *GFP* gene suppression can be detected by the unmasking of red fluorescence emitted from chlorophyll. We observed that *GFP* silencing was visible under UV light on treated leaves as soon as 2 days after treatment, and phenotype on treated leaves was maximal by 8 days after treatment (Fig. 1a). Leaves treated with a control dsRNA (24 nt of the 5-enolpyruvylshikimate-3-phosphate synthase (*EPSPS*) coding sequence from *Amaranthus palmeri*) did not show development of red fluorescent spots. Systemic silencing in veins of upper leaves was strong at 13 days after treatment in this experiment and continued to progress during the life of the plant. Quantification of an RNA gel blot showed that expression of the *GFP* transgene in treated leaves was reduced 25% 1 day after treatment and 67% 2 days after treatment (Fig. 1b).

**Fig. 2:**
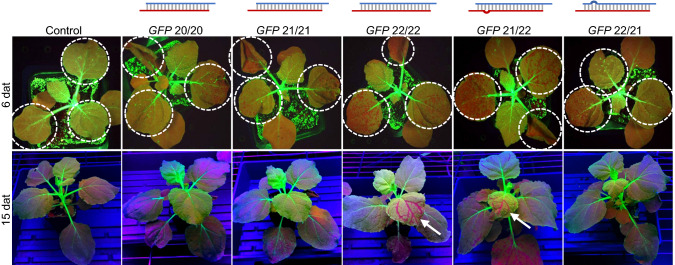
An siRNA with 22 nt in the guide strand was required for systemic *GFP* silencing in *N. benthamiana*. siRNA (25 μl siRNA at 1 μg/μl in 0.01% Silwet L77) was applied onto true leaves 3, 4, and 5 (circled) of 16-day old plants followed by abrasion with #600 grit sandpaper (3 plants per treatment). siRNAs are diagrammed as guide (red) and passenger (blue) strands, and base pairings indicated by grey vertical bars. A loop is shown where an unpaired base is present (*GFP* 21/22 and *GFP* 22/21). siRNA sequences are shown in Table 1. Plants were photographed under strong blue lights (for 6 dat photos) and UV lights (for 15 dat photos). One representative plant per treatment is shown and treated leaves are circled. Arrows point to leaves showing systemic *GFP* silencing. Control, 24 nt dsRNA from *A. palmeri EPSPS*

To better understand the sRNA structural requirements for optimal activity, we treated 16-day old *N. benthamiana* leaves 3, 4 and 5 (counted from the base of the plant) with siRNAs targeting *GFP* from 20 to 22 nt, including hybrid molecules containing one strand with 22 nt and the other with 21 nt (Table 1). Plants were examined at 6 days after treatment under blue light to observe *GFP* silencing phenotypes (Fig. 2). Note that the treated leaves are circled, and red spots on those leaves indicate silencing of the *GFP* transgene. We consistently observed that leaves 1 and 2 appeared red without siRNA treatment and so they were not used in the experiments. We hypothesize that the *GFP* transgene is not strongly expressed in leaves 1 and 2 at this developmental stage. However, untreated leaves 3 and older consistently showed green GFP fluorescence and silencing of *GFP* in leaves treated with *GFP* siRNAs could be observed by appearance of red spots in these leaves. While some silencing was evident at 6 days after treatment from all *GFP* siRNAs tested in this experiment from 20 to 22 nt, silencing in treated leaves was strongest with the 22/22 or 21/22 configurations, which both included the 22 nt sequence in the guide strand of the siRNA. Systemic silencing was observed at 15 days after treatment in plants that had been treated with siRNAs containing a 22 nt guide strand, and both 22/22 and 21/22 siRNA configurations produced systemic silencing (Fig. 2). A sequence containing 22 nt in the passenger strand with a 21 nt guide strand (22/21) did not induce systemic silencing (Fig. 2).

**Fig. 3:**
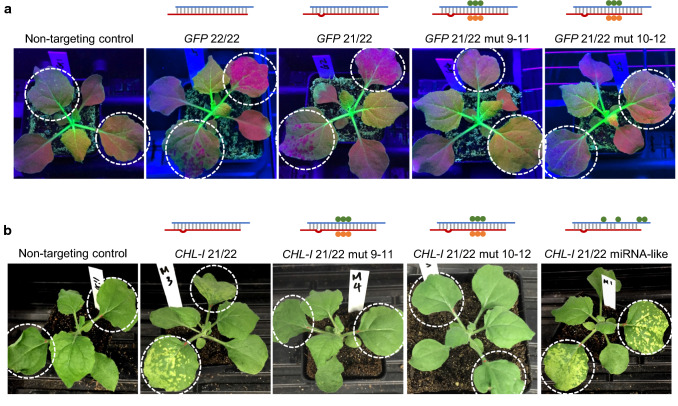
Activity of siRNAs with slice site mutations was consistent with an RNAi mode-of-action. dsRNA (20 μl dsRNA at 1 μg/μl in 0.01% Silwet L77) was applied and evenly spread onto leaves 3 and 4 of 14-day plants followed by abrasion with #600 grit sandpaper (2 plants per treatment). The treated leaves are circled. siRNAs are diagrammed as guide (red) and passenger (blue) strands, and base pairings indicated by grey vertical bars. A loop is shown where an unpaired base is present (*GFP* 21/22 and *CheI* 21/22). Green or orange dots indicate mutated bases, and mutations at positions 9–11 or 10–12 target the presumptive slice sites. siRNA sequences are shown in Table 1. **a** A representative plant treated with *GFP-*targeting or control siRNAs. The plants were photographed under UV lights at 5 dat. **b** A representative plant treated with *MgCheI-*targeting or control siRNAs. The plants were photographed under white LED lights at 5 dat. Control, 24 nt dsRNA from *A. palmeri EPSPS*

To further confirm *GFP* silencing observed with the abrasion method was a result of RNAi, siRNAs containing mutations of the predicted slice site were tested (Table 1). Consistent with topically delivered siRNAs acting in the RNAi pathway, mutations of the *GFP* siRNAs from 9–11 (GGC to CCG) or 10–12 (GCU to CGA) nt, which would alter the predicted AGO slice site, greatly decreased silencing activity (Fig. 3a).

### Silencing of the endogenous *magnesium cheletase I (CHL-I)* gene in *N. benthamiana*

We then targeted an endogenous gene in *N. benthamiana* encoding *magnesium cheletase I (CHL-I)*, which is involved in chlorophyll biosynthesis (Kruse et al. 1997). Several different *CHL-I* 21/22 siRNAs were tested with sandpaper delivery in *N. benthamiana* line 16C, and the most efficacious sequence was used in further experiments (data not shown; Table 2, CheI_21/22). Silencing of *CHL-I* resulted in appearance of yellow spots where RNA had been delivered (Fig. 3b). 22 nt variants of the 21/22 *CHL-I* siRNA were made containing mutations of nucleotides at positions 9–11 (UCU to AUC) or 10–12 (CUC to UCG) of the guide strand to inactivate the predicted AGO slice site. These siRNAs were not active in silencing *CHL-I* using sandpaper-based dsRNA delivery, supporting an RNAi mode-of-action for this delivery method (Fig. 3b). An siRNA containing mismatches in the passenger stand (positions 9, 13, and 19) was created to mimic a miRNA structure (Table 2 and Fig. 3b, “*CHL-I* 21/22 miRNA-like”) (Jones-Rhoades and Bartel 2004). This structure had activity comparable to the 21/22 siRNA that included complete sequence identity to the *CHL-I* target on both strands (Fig. 3b, “*CHL-I* 21/22”). These results are all consistent with the hypothesis that silencing of *CHL-I* with topically-applied siRNA occurs through the RNAi pathway. Plants were observed for 2 weeks after treatment with *CHL-I* siRNAs, but systemic silencing was never observed for this gene. In contrast, at this same time point systemic silencing for the *GFP* transgene was observed for plants treated with GFP siRNA (Figs. 1, 2).

**Table 2:** siRNAs targeting the *N. benthamiana CHL-I* gene

Duplexes	Sense (passenger) strand (5′–3′):antisense (guide) strand (5′–3′)	Figure/Table
***Magnesium cheletase I***		
* CHL-I*_21/22	GGG-CCGUGAGAGAUGCAGAAA:UCUGCAUCUCUCACGGUCCCCA	Figure 3
* CHL-I*_21/22_mut 9–11	GGG-CCGUG**GAU**GAUGCAGAAA:UCUGCAUC**AUC**CACGGUCCCCA	Figure 3
* CHL-I*_21/22_mut 10–12	GGG-CCGU**CGA**AGAUGCAGAAA:UCUGCAUCU**UCG**ACGGUCCCCA	Figure 3
* CHL-I*_21/22_miRNA-like	GGG-CCGUG**U**GAG**U**UGCAG**UG**A:UCUGCAUCUCUCACGGUCCCCA	Figures 3, 4; Table 3
* CHL-I*_22/22_miRNA-like	GGGACCGUG**U**GAG**U**UGCAG**UG**A:UCUGCAUCUCUCACGGUCCCCA	Figure 4; Table 3
* CHL-I*_21/21_miRNA-like	GGACCGUG**U**GAG**U**UGCAG**UG**A:UCUGCAUCUCUCACGGUCCCC	Figure 4; Table 3
* CHL-I*_22/21_miRNA-like	GGA**U**CCGUG**U**GAG**U**UGCAG**UG**A:UCUGCAUCUCUCACGG-UCCCC	Figure 4; Table 3
* CHL-I*_21/21	GGACCGUGAGAGAUGCAGAGC:UCUGCAUCUCUCACGGUCCCC	Figure 5
* CHL-I*_22/22	GGGACCGUGAGAGAUGCAGAGC:UCUGCAUCUCUCACGGUCCCCA	Figure 5, Table 5

The two strands are separated by a colon. Bold letters indicate introduced mutations or mismatches from the antisense strand sequence

### Transitive sRNA production after topical treatment with siRNAs

**Fig. 4:**
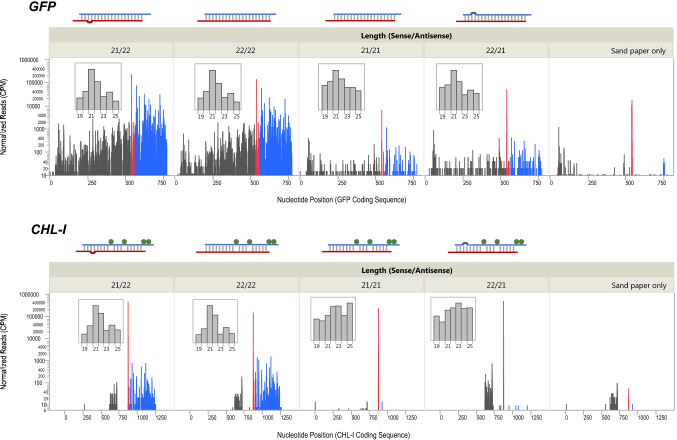
Transitive sRNA production was greater when the guide strand of the siRNA contained 22 nucleotides. siRNAs were applied to the 2 youngest partially expanded leaves of 3-week old plants (3 reps), and treated leaves were lightly abraded with sandpaper. The applied siRNAs are diagrammed above each panel with guide (red) and passenger (blue) strands, and base pairs shown as grey bars. A loop indicates an unpaired base, and green dots indicate bases mutated to mimic a miRNA structure. Four days after treatment phenotypic tissue was harvested from the 2 plants with the best phenotypes and RNA was isolated and sRNA sequencing was conducted. Sense and antisense sRNAs 19–25 nt in length were mapped to the targeted transcripts (*GFP* or *CHL-I*). The red bars indicate sense and antisense reads mapping to the site corresponding to the applied siRNA. Black bars represent sRNAs mapped 5′ to the applied siRNA and blue bars represent reads mapped 3′ to the applied siRNA. CPM, counts per million. Insets show distribution of sRNA sizes

Since 22 nt miRNAs have been associated with transitive sRNAs or phasiRNAs (Axtell 2013), we examined sRNAs present in tissue showing a silencing phenotype for *GFP* or *CHL-I* after treatment with siRNAs containing a 21 or 22 nt sequence as either the guide or passenger strand, or both (Tables 1, 2). The base siRNA for *CHL-I* was the “miRNA-like form” demonstrated to have activity comparable to the simple 21/22 siRNA (Fig. 3b, Table 2), while the base *GFP* siRNA was a 22/22 siRNA. Three-week old plants were treated with siRNAs using sandpaper abrasion. Silencing phenotypes were observed but not quantified in this experiment, and all siRNA treatments displayed local silencing phenotypes as previously observed for these siRNAs. Leaf tissue was harvested for sRNA analysis from the two plants with the strongest silencing phenotypes for each siRNA 4 days after treatment. Because of limitations in sequencing resources, the 2 samples from each treatment were pooled for sequencing. sRNAs were mapped to the mRNA sequences for the targeted genes. A baseline level of sRNAs for the *GFP* transgene and for *CHL-I* were observed in a control that involved application of buffer followed by sandpaper rolling (Table 3). Some contamination with the applied siRNA sequence was noted in this control treatment (Fig. 4, red bars). We concluded that this contamination occurred at a point after delivery of applied siRNAs, since its presence was not associated with any silencing phenotype. Treatment with a 22 nt siRNA in the guide strand targeting *GFP* (21/22 or 22/22) resulted in very high levels of transitive sRNAs, from 1000 to 1600 times the control level (Table 3). sRNA levels were elevated both 5′ and 3′ to the siRNA binding site, but the amount of sRNAs was greatest 3′ of the siRNA binding site (Table 3 and Fig. 4). siRNAs targeting *GFP* containing 21 nt in the guide strand, even with 22 nt in the passenger strand (22/21 or 21/21), induced relatively low levels of transitive sRNA production, about 14 times the control. Thus, the 22 nt siRNA targeting *GFP* resulted in approximately 75 times more transitive sRNAs 3′ to the siRNA binding site compared to the 21 nt siRNA. The transitive sRNAs produced in response to any siRNA were mostly 21 nt in length, but some 22 and 24 nt sRNAs were also produced (Fig. 4 inset graphs). No significant differences in sRNA size ratios were associated with a particular siRNA configuration.

**Table 3:** sRNAs mapping to *GFP* and *CHL*-*I* mRNAs after treatment of leaves of *N. benthamiana* with siRNAs of different structures

Applied siRNA length (nt) (passenger/guide strands)	sRNA position relative to applied siRNA	Total sense and antisense small RNAs 19–25 nt (CPM)	
		*GFP*	*CHL-I*
21/22	5'	84,701	1101
	3'	449,730	7208
22/22	5'	58,392	1336
	3'	294,609	14,175
21/21	5'	4410	143
	3'	3998	14
22/21	5'	6170	4450
	3'	4148	39
Sandpaper only	5'	3206	866
	3'	281	11

*CPM* counts per million

The *CHL*-I gene also produced transitive sRNAs in response to targeting 22 nt siRNAs but the response was much weaker than for the *GFP* transgene, even though the tissue harvested had strong gene knock-down as observed by the chlorotic phenotype. For the *CHL-I* target, only a low level of transitive sRNAs were generated in response to treatment with siRNAs containing 22 nt in the guide strand (21/22 or 22/22), and these sRNAs were only produced 3′ to the siRNA binding site (Table 3 and Fig. 4). No increase in transitive sRNAs 3′ to the siRNA binding site was observed when the siRNA had 21 nt in the guide strand. Comparing the response to 22 nt siRNAs, about 20 times more transitive sRNAs 3′ to the siRNA binding site were produced for *GFP* compared to *CHL-I*. The size distribution of *CHL-I* transitive sRNAs were similar to those produced for *GFP*, with 21 nt having the greatest representation, but 22 and 24 nt sRNAs were also made (Fig. 4 inset graphs). This size distribution was not observed when the siRNAs had 21 nt guide strands, consistent with the lack of transitive sRNA production.

### Production of higher levels of transitive sRNAs in response to 22 nt siRNAs was associated with greater silencing activity

To expand our understanding of silencing of endogenous genes by topically-applied dsRNAs, we targeted two other endogenous genes in *N. benthamiana* that when silenced produce chlorotic tissue. These genes were *magnesium cheletase-H* (*CHL-H*), another sub-unit of the magnesium cheletase enzyme (Kruse et al. 1997), and *GENOMES UNCOUPLED4 (GUN4)*, a gene involved in plastid retrograde signaling (Larkin et al. 2003). While topical treatment with 22 nt siRNAs targeting these genes (Table 4) produced yellow spots in the treated leaves, systemic silencing was not observed (data not shown).

**Table 4:** siRNAs targeting *GUN4* and *CHL-H* genes

Duplexes	Sense (passenger) strand (5′–3′):antisense (guide) strand (5′–3′)	Figure/Table
*N. benthamiana GUN4*		
*GUN4*_21/21	CAGGAGAAGCAGCAGUUAAAC:UUAACUGCUGCUUCUCCUGCU	Figure 5
*GUN4*_22/22	GCAGGAGAAGCAGCAGUUAAAC:UUAACUGCUGCUUCUCCUGCUA	Figure 5, Table 5
*N. benthamiana CHL-H*		
*CHL-H*_21/21	CGAAGGAGUUAUGCGAAUACC:UAUUCGCAUAACUCCUUCGUU	Figure 5
*CHL-H*_22/22	ACGAAGGAGUUAUGCGAAUACC:UAUUCGCAUAACUCCUUCGUUU	Figure 5, Table 5
*A. cruentus CHL-H*		
*CHL-H*_21/21	CCCUCAACAAAACUUCUGAAU:UCAGAAGUUUUGUUGAGGGUU	Figure 6
*CHL-H*_22/22	ACCCUCAACAAAACUUCUGAAU:UCAGAAGUUUUGUUGAGGGUUU	Figure 6

The two strands are separated by a colon

**Fig. 5.**
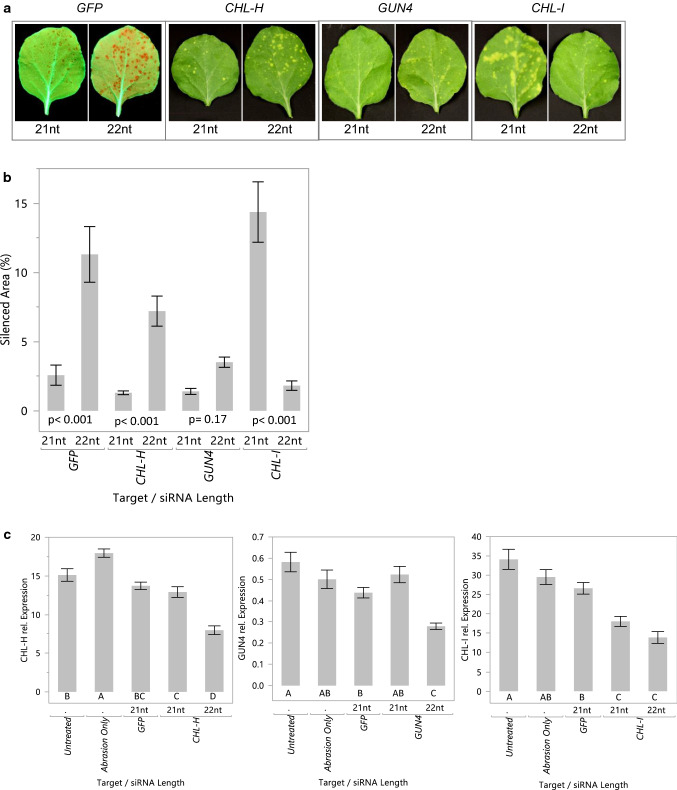
22 nt siRNAs often result in greater local silencing compared to 21 nt siRNAs. **a** For each gene target, equal concentrations of 21 or 22 nt siRNAs were applied with sandpaper abrasion to the third leaf of 21–22 day old *N. benthamiana* plants. Plants were photographed under white (*CHL-I*, *CHL-H*, *GUN4*) or UV (*GFP*) light 7 days after treatment. *N* = 4 for *GFP* and *N* = 6 for other gene targets. A leaf with a representative phenotype is shown. **b** Silenced area for these treatments was quantified using Image J. **c** Plants (8 per treatment) were treated as described above with siRNAs against endogenous genes, and phenotypic tissue was harvested 5 days later for RNA isolation and quantification of targeted mRNAs by RT-PCR. All data are means ± standard error. *P* values for contrasts comparing silenced area produced using 21nt to 22nt siRNA are provided. Letters under the bars indicate significant difference by Student’s *t* test, *α* < 0.05

An experiment was conducted to determine whether 21 vs 22 nt siRNAs had differential effects on gene silencing within the treated leaves. 21 or 22 nt siRNAs targeting *GFP*, *CHL-I*, *CHL-H* and *GUN4* were delivered to leaves of *N. benthamiana* line 16C at a dose that allowed separation of phenotypic spots. 22 nt siRNAs showed greater activity than 21 nt siRNAs in generating silencing phenotypes for *GFP*, *CHL-H*, and *GUN4*, and for these genes the phenotype was increased approximately 4.4-, 5.5- and 2.5-fold, respectively (Fig. 5a, b). However, the 21 nt siRNA targeting *CHL-I* produced 7.9 times greater silencing phenotype than the 22 nt siRNA. These effects were confirmed in multiple experiments. mRNA levels for the targeted endogenous genes were measured in phenotypic spots from these treatments and compared to mRNA levels from green tissue of controls. 22 nt siRNAs had greater activity than 21 nt siRNAs in reduction of mRNA levels for *GUN4* and *CHL-H* (Fig. 5c). However, 21 and 22 nt siRNAs had similar activity in reducing mRNA levels for *CHL-I*.

sRNA sequencing was carried out to examine the relative levels of transitive sRNAs produced in response to 22 nt siRNAs for these endogenous genes. siRNAs for *CHL-I*, *CHL-H* and *GUN4* (Tables 2, 4; 1 μg/μl) were applied to the 2 youngest leaves of 3-week old *N. benthamiana* line 16C plants. Phenotypic tissue from the treated leaves was harvested 7 days after treatment. RNA was extracted from this tissue and processed for sRNA sequencing. The background levels of sRNAs for a given gene can be estimated by the number of transitive sRNAs produced in the absence of the targeting siRNA (Table 5). When targeting *CHL-H* with a 22 nt siRNA, a very high number of transitive sRNAs were produced (Table 5). As seen before (Table 3), only low levels of sRNAs were observed when targeting *CHL-I*. A low but significant number of transitive sRNAs were observed for the *GUN4* target (Table 5). In each of these cases, transitive sRNAs mapped 3' to the applied siRNA binding site. The ratio of transitive siRNAs for these genes after treatment with a 22 nt siRNA was approximately 32: 8: 1 (*CHL-H* > *GUN4* > *CHL-I*).

**Table 5:** sRNAs mapped to mRNAs of targeted endogenous genes in *N. benthamiana* after treatment with 22 nt siRNAs. CPM, counts per million

22 nt siRNAs	Total 19–25 nt sense and antisense sRNAs 3′ to the applied siRNA (CPM)		
	*CHL*-*I*	*CHL*-*H*	*GUN4*
*CHL-I*	7419	502	0
*CHL-H*	11,439	1,834,785	39
*GUN4*	456	1674	58,009

### Topical RNAi suppression of *CHL-H* gene in *Amaranthus cruentus*

**Fig. 6:**
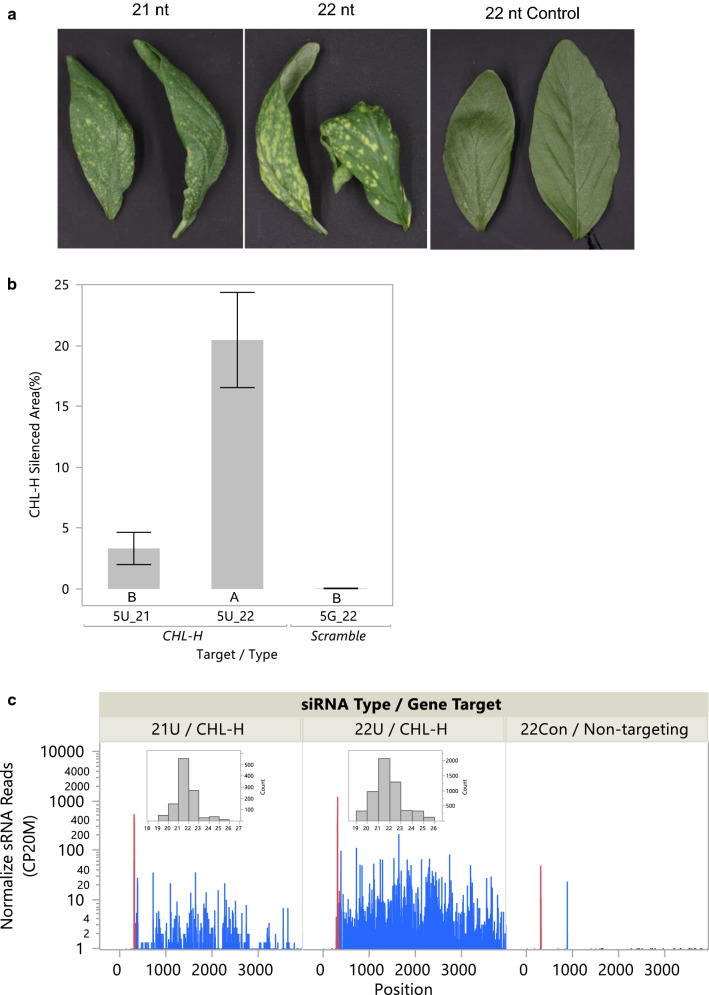
Topical treatment of *A. cruentus* leaves with siRNAs targeting *CHL-H* showed that 22-mers produced a stronger phenotype compared to 21-mers and resulted in more transitive sRNAs. siRNAs were applied to 14-day-old plants (6 reps), and treated leaves were abraded with dry particles. **a** treated leaves were photographed 7 days after treatment, and leaves with representative phenotypes for the treatment are shown. **b** Leaf silenced area was quantified using Image J, and plotted with standard errors. Letters under the bars indicate significant difference by Student’s *t* test, *α* < 0.05. **c** small RNAs (19–25 nt) were mapped to the targeted *CHL-H* transcript. The red bars indicate sense and antisense reads mapping to site corresponding to the applied siRNA. Blue bars represent sense and antisense small RNA reads mapped 3′ to the applied siRNA. CP20M, counts per 20 million

To determine whether topically applied 22 nt siRNAs have similar properties in other species, we chose to target *CHL-H* in *Amaranthus cruentus*, a model for important weed species including Palmer Amaranth *(A. palmeri)* and Waterhemp *(A. tuberculatus)*. Similar to what was observed in *N. benthamiana*, a 22 nt siRNA was more effective in silencing *CHL-H* than was a 21 nt siRNA in *A. cruentus* with about 5 times more silenced area (Fig. 6a, b). The background levels of sRNAs for the *CHL-H* transcript in leaf tissue treated with a non-targeting dsRNA were very low, but significant numbers of transitive sRNAs were produced in response to 21 and 22 nt siRNAs, and these sRNAs mapped 3' to the applied siRNA binding site (Table 6 and Fig. 6c). The level of transitive sRNAs produced in response to the 22 nt siRNA was about 12 times greater than those produced for a 21 nt siRNA. The sizes of transitive sRNAs for *CHL-H* in *A. cruentus* were mostly 21 nt with a significant number of 22 nt sRNAs also (Fig. 6c insets). These results show that 21 and 22 nt siRNAs have similar effects in both *A. cruentus* and in *N. benthamiana*. In both species, 22 nt siRNAs targeting *CHL-H* were more active than 21 nt siRNAs in producing a greater silencing phenotype and also by inducing a greater number of transitive sRNAs for many gene targets.

**Table 6:** Sum of sRNAs mapping to the *CHL-H* mRNA after treatment of *A. cruentus* with 21 or 22 nt siRNAs

Applied siRNA length (nt)/gene target	sRNA position relative to applied siRNA	Total sense and antisense small RNA 19-25nt (CPM)
21/*CHL-H*	5′	5
	3′	1862
22/*CHL-H*	5′	41
	3′	22,679
22/non-targeting		927

## Discussion

To better understand gene suppression mechanisms in plants we’ve developed a method for delivery of specific siRNAs into cells of intact leaves by topical application of these RNAs followed by gentle mechanical abrasion. Using this method we were able to demonstrate RNAi silencing phenotypes for the *GFP* transgene and 3 nuclear-encoded genes involved in chlorophyll biosynthesis or plastid signaling in *N. benthamiana* (*CHL-I, CHL-H* and *GUN4)* and for *CHL-H* in *A. cruentus*. siRNAs from 20 to 22 nt were active in silencing a *GFP* transgene in the treated leaf tissue of *N. benthamiana* (Fig. 2), and mutations of the predicted slice site reduced or eliminated silencing activity for both *GFP* and *CHL-I* (Fig. 3). Thus, the properties of siRNAs active in silencing these gene targets with this method were consistent with an RNAi mode-of-action.

Strong systemic silencing of the *GFP* transgene was achieved with the abrasion method when the *GFP*-targeting siRNA contained a 22 nt guide strand, and both 22/22 and 21/22 nt siRNAs had equivalent efficacy (Fig. 2). These results agree with reports using a high-pressure spray to deliver dsRNAs to silence *GFP* in the 16C line (Dalakouras et al. 2016). We also showed that the 22 nt sequence must be present in the guide strand, as a 22/21 structure containing a 22 nt passenger strand, was not effective in generating a systemic response.

To determine whether systemic silencing could be initiated for endogenous genes using topically applied siRNAs, we targeted mRNAs for 2 sub-units of magnesium cheletase and the *GUN4* gene. Strong silencing phenotypes could be observed for each of these gene targets in the treated leaves (Figs. 3b, 5, 6) but no systemic silencing was observed for any of them. It was recently shown that systemic silencing of the *GFP* transgene in *N. benthamiana* was associated with high levels of transgene expression (Hendrix et al. 2021), and perhaps the endogenous genes tested weren’t expressed to a sufficiently high level to induce a systemic silencing response. In addition, other characteristics of these genes may have contributed to the lack of systemic silencing. For example, a distinctive feature shared by the endogenous genes tested is that they encode proteins that localize to the plastid, and they were chosen for this work to take advantage of their visible silencing phenotypes. However, it is possible that genes associated with plastids may not be representative of all endogenous genes with regard to potential for systemic silencing. For example, there is evidence that mRNAs for some genes involved in plastid function, including *CHL-H*, associate with plastids (Gibson et al 1996; Marrison et al 1996), and perhaps this compartmentalization of the mRNA within the cytoplasm limits participation in pathways required for a systemic silencing response. Testing genes involved in a variety of different cellular pathways could identify instances of a systemic silencing response for some endogenous genes. In addition, other features of genes that permit systemic silencing could be explored with this RNA delivery method.

Secondary transitive siRNAs can be produced in response to miRNAs or siRNAs and may amplify a silencing signal (Moissiard et al. 2007; Axtell 2013). We previously showed that transitive siRNAs were produced in treated leaves of *N. benthamiana* line 16C after silencing the *GFP* transgene or the *CHL-H* gene using a carbon dot formulation to deliver 22 nt siRNAs targeting these genes (Hendrix et al. 2021). In comparing different *GFP*-expressing lines, that study found that high transgene expression level and transitive sRNA abundance in the application leaves were associated with systemic silencing. *GFP* sRNAs were also observed in systemic leaves and their amount was weakly correlated with the amount of *GFP* sRNAs in treated leaf tissue. Abundant systemic *GFP* sRNAs were only found in systemically silenced lines, suggesting a role for secondary sRNAs in systemic silencing. To determine whether transitive siRNAs were produced in response to siRNAs delivered with the abrasion method, and to learn more about their roles in the strength of a local response or in systemic silencing for different genes, we examined sRNAs in leaves treated with various siRNAs.

Sequencing of sRNAs from tissues treated with *GFP* siRNAs showed that a 22 nt RNA guide strand resulted in production of high levels of *GFP* transgene secondary sRNAs (Table 3 and Fig. 4). In contrast, a 22 nt RNA in the passenger strand did not induce transitive siRNA production. This result is in conflict with a previous report which found that having a 22 nt only in the passenger strand was sufficient to induce accumulation of secondary siRNAs (Manavella et al. 2012). We found good correspondence between different siRNAs that induced high levels of secondary sRNAs and those that produced systemic silencing activity for the *GFP* transgene in *N. benthamiana*. This result is consistent with the findings of Hendrix et al. (2021) which showed that *GFP* transgene-expressing events with systemic silencing also had high levels of transitive sRNAs.

The mechanism for how 22 nt siRNAs or miRNAs act to generate secondary sRNAs while 21 nt forms do not is not known, but it has been suggested that binding of a 22 nt miRNA with AGO1 may alter the structure of AGO1 so that it can recruit proteins such as RDR6, which are necessary for secondary sRNA production (Chen et al. 2010; Cuperus et al. 2010).

sRNA sequencing showed that 22 nt siRNAs induced some transitive sRNA production for each of the endogenous genes examined (Figs. 4, 6, Tables 5, 6). However, the amount of secondary sRNAs was quite different for the different gene targets. Only a very low level of secondary sRNAs was produced in response to a 22 nt siRNA for *CHL-I* (Tables 3, 5), and this response was only observed with a 22 nt siRNA and not with a 21 nt siRNA. Somewhat higher levels of secondary sRNAs were produced for the *GUN4* mRNA after treatment with a 22 nt siRNA, but very high levels of secondary sRNAs were produced for the *CHL-H* mRNA in *N. benthamiana* (Table 5). Similarly, high levels of secondary sRNAs were also observed for *CHL-H* in *A. cruentus* after targeting with a 22nt siRNA (Table 6; Hendrix et al. 2021). Despite these high levels of secondary sRNAs for *CHL-H*, no systemic silencing was observed for this gene in either species. Since we didn’t compare secondary sRNAs for *GFP* and *CHL-H* in the same experiment, it’s not possible to address whether a threshold of transitive sRNA concentration exists that is required to initiate systemic silencing. Published reports of systemic silencing have focused on examples using transgenes, either stably integrated or transiently expressed. In all of these cases very high levels of target and siRNAs or amiRNAs would be present.

While the sRNA sequencing results would have greater reliability with a more robust replication strategy, there was considerable consistency among the results that provided confidence in the conclusions. For example, comparison of transitive sRNA production in response to treatment with 21 or 22 nt siRNAs in the guide strands of siRNAs targeting either *GFP* or *CHL-I* involved two related siRNAs for each size class, and these siRNA pairs performed very similarly with respect to the numbers of secondary sRNAs produced (Table 3, Fig. 4). Furthermore, a consistent pattern of greater induction of transitive siRNAs with 22 mers compared to 21 mers was observed across genes and species (Tables 3, 6, Figs. 4, 6).

The distribution of secondary sRNAs along the coding sequence were different for the *GFP* transgene compared to the endogenous genes examined in this study. For *GFP*, secondary sRNAs were produced both 3′ and 5′ to the targeting siRNA (Fig. 4 and Table 3). However, for *CHL-I* (Fig. 4 and Table 3) and for *CHL-H* (Fig. 6 and Table 6) sRNAs induced by the treatment were mostly observed 3′ to the targeting siRNA site. It’s possible that presence of secondary sRNAs 5′ to the targeting siRNA could be associated with systemic silencing, but additional examples are needed to make this conclusion.

Although production of secondary sRNAs with 22 nt siRNAs did not result in systemic silencing of endogenous genes, an increase in the amount of local silencing was observed for both the endogenous *CHL-H* and *GUN4* targets as well as for the *GFP* transgene when 22 nt siRNAs were used (Figs. 5, 6). We hypothesize that the secondary sRNAs move cell-to-cell as has been described for other sRNAs (Melnyk et al. 2011; Liu and Chen 2018) and that some of them are active in gene silencing in these neighboring cells.

The response of the *CHL-I* gene to siRNAs of different sizes was different from the other genes tested, since for *CHL-I* the silencing phenotype was greater with the 21 nt siRNA. It is possible that the 22 nt siRNA had reduced silencing efficacy for *CHL-I* due to its very weak induction of secondary sRNAs for this gene target (Tables 3, 5), but in the absence of such signal amplification one might expect equivalent activity of the 22 nt and 21 nt forms. In fact, reduction of *CHL-1* mRNA was equivalent for 21 and 22 nt siRNAs. One mechanism that could explain these findings would be if the 21 nt siRNA had activity to suppress translation of the *CHL-1* mRNA as well as to slice the mRNA. Both activities are established mechanisms for RNAi (Arribas-Hernández et al. 2016; Li et al. 2013; Fang and Qi 2016). In that case, mRNA concentrations could be equally reduced with the two siRNAs but the 21 nt siRNA would produce a stronger silencing phenotype. The weak induction of secondary small RNAs for *CHL-I* may be an intrinsic property of this gene. Chen et al. (2010) noted that 22 nt miRNAs do not trigger secondary siRNA production for all genes. Additional research to understand the basis for the different responses of the genes in our study may generate useful insights about the mechanism of secondary sRNA production.

The sandpaper abrasion method for introduction of sRNAs into plant cells is easy to use, highly reproducible, and results in RNAi silencing of the targeted genes. Although plant tissues are known to contain abundant RNAses in both the apoplast and within the cell (Green 1994; Pérez-Amador et al. 2000), we found specific effects of 21 vs 22 nt siRNAs, suggesting that at least some of the siRNAs are preserved intact during uptake into cells. This method can be used for additional investigation of RNAi mechanisms in plant cells, and may have utility in certain cases for production of traits of agronomic interest.

## Materials and methods

### Plant materials

The *N. benthamiana* line 16C expressing a *GFP* transgene (Ruiz et al. 1998) was obtained from David Baulcombe (Ruiz et al. 1998). Plants were grown in a growth chamber under a 16 h day: 8 h night photoperiod. Light intensity was approximately 250 μmol m^−2^ s^−1^ and temperatures were 26 °C during the day and 18 °C at night.

*Amaranthus cruentus* seed was obtained from Johnny’s Selected Seed (https://www.johnnyseeds.com/). Plants were grown at 25 °C with 16 h day length and 100 μmol m^−2^ s^−1^ light intensity. Seeds were germinated on coconut coir plugs (Jiffy Products of America, Inc., Lorain, OH) and then transplanted on day 7 into 2.5 inch pots filled with Berger BM2 media. Plants were irrigated daily with an ebb and flow system with Peters 20-20-20 liquid fertilizer.

### siRNA and gene sequences

All siRNAs (20–22 nt) were synthesized by IDT (Integrated DNA Technologies, Inc., Iowa, USA) with 2 nt 3′ overhangs. An algorithm based on Reynolds score was used to select siRNAs for screening, as described in Yang et al. (2021). No RNA base or linkage modifications were introduced during the synthesis. Sequences are provided in tables within the text.

Accession numbers for *N. benthamiana* and *A. cruentus* genes silenced in these studies are listed in Supplementary Table S1.

### Abrasion-based delivery of siRNAs to plant leaves

#### *N. benthamiana*

Typically, 20–25 μl of a solution of 1 μg/μl siRNA in water or 0.01–0.05% Silwet L77 was pipetted onto the adaxial surface of 2–3 partially expanded leaves of 14–21-day-old *N. benthamiana* plants. Care was taken to ensure complete coverage of the leaf surfaces. After the solution dried, the adaxial surface of the leaf was lightly abraded using 600grit sandpaper affixed to a wooden dowel (7 cm long, 1.2 cm diameter). The abrasion was achieved with a gently rolling motion on the treated leaf surface using support from a gloved finger on the abaxial side of the leaf. The goal of this procedure was to lightly abrade the adaxial surface of the leaf without crushing or tearing the leaf. The leaves may wilt after abrasion but will recover if inner leaf tissues are not damaged to point of cell lysis and death.

For the experiment shown in Fig. 5, the concentration of each 22 nt siRNA that allowed resolution of individual phenotypic spots was determined empirically. Then, equal concentrations of 21 and 22 nt siRNAs for a gene target were compared for activity. siRNA concentrations used were: *CHL-H* (0.5 μg/μl); *GUN4* (0.5 μg/μl); *GFP* (1 μg/μl); *CHL-I* (0.2 μg/μl).

#### *Amaranthus cruentus*

The manual abrasion method described for *N. benthamiana* was efficacious for *A. cruentus*, but the subjective nature of the method was not conducive to rigorous quantitative comparisons of siRNAs or formulations. A more controlled method using a custom-built dry particle sprayer was developed to address this need. Here, dsRNA was dissolved at 3 mg/ml (21/22 mers) or 6 mg/ml (66 mer) in a solution containing 50 mM sucrose, 5 mM MES pH 5.7, 0.08% L77 Silwet. The dsRNA solution (8–10 μl) was applied to each of the L3, L4 and terminal leaves and allowed to air dry. The leaves were then abraded with 220 mesh silicon carbide particles (Kramer Industries) using a custom-built dry particle sprayer equipped with a 95015-TC spray tip (Spraying Systems Co.) at a pressure of 45 psi, a forward track speed of 36.5 cm/s, set 15 cm from the leaf surface.

### Phenotype image capture and analysis

For young plants GFP fluorescence was excited using a high intensity blue LED light source (465 nm, Photon System Instruments) and imaged using a Canon EOS 70D camera with 58 mm Tiffen Green #11 and Yellow #12 filters. GFP fluorescence of older plants was visualized using a UV light and images captured using an iPhone 7.

Phenotypes were quantified using the Image J 1.51a platform (National Institutes of Health, USA). The threshold color panel was used to distinguish phenotypic regions of leaves. Both the leaf border and the phenotypic borders were manually outlined. Then, the program automatically quantitated the pixel number within the whole leaf or the phenotypic region, and silenced area was represented by the ratio of the phenotypic area pixels to the total leaf area pixels. Color threshold settings were kept uniform within experiments.

Photographs used for figures were cropped and adjusted for brightness, contrast, hue and saturation using Paint.net. All images from an experiment were adjusted using the same settings.

### Sample collection and RNA purification

Leaf discs were collected from treated leaves using 5 mm biopsy punches (Integra, York, PA) and were immediately frozen on dry ice and stored at − 80 °C. Total RNAs were extracted from 10 disks per sample using Trizol reagent (Invitrogen, CA, USA) following the instructions of the product manual.

### RNA gel blot

The RNA gel blot was done by using the DIG Northern Kit (Roche, Mannheim, Germany). The blot was probed with part of the *GFP* transcript (Supplementary Table S2).

### RNA detection by RT-qPCR

Frozen leaf disc samples were ground with ball bearings and extracted using Direct-zol™-96 RNA (Zymo Research; Irvine, CA, USA). Total RNA was eluted in DNAse/RNAse free water, quantified to ensure the concentration was less than 200 ng/μl and used directly for cDNA synthesis. Oligo dT cDNA was generated with High Capacity cDNA Reverse Transcription Kit (Applied Biosystems; Foster City, CA, USA) per the manufacturer’s protocol. Singleplex qPCR was performed with a 4-fold dilution of cDNA using either Quanta Bio (Beverly, MA) PerfeCTa Fast Mix II for probe based assays or PerfeCTa SYBR green Supermix and run on BioRad CFX96 Real-Time PCR Detection System (Hercules, CA, USA). For quantification, *EF1a* and *UKN1* were used as internal normalizer genes. Relative Quantity (RQ) was calculated by 2^−(Gene-of-interest Ct − GeoMean Internal Normalizer Ct). Probe sequences are shown in Supplementary Table S3.

### sRNA sequencing and analysis

Illumina’s TruSeq small RNA Library Preparation Kit was used to prepare small RNA libraries following the manufacturer’s protocol (Document # 15004197v02) with slight changes to the library purification steps. After cDNA amplification, uniquely indexed libraries were pooled together in equimolar amounts and size separated on a 6% Novex TBE PAGE Gel. The gel was stained for twenty-minutes with 1× SYBR Gold. A minimum of 3 million reads per size-selected library was generated using Illumina’s NextSeq platform.

To assess quality of the sequencing libraries, fastqc was used (Andrews 2010, http://www.bioinformatics.babraham.ac.uk/projects/fastqc), and for read-quality filtering and trimming adapters, Trimmomatic was used (Bolger et al. 2014). Read processing and mapping was performed with SAMtools (Li et al. 2009), BAMtools (Barnett et al. 2011), and bowtie2 using very-sensitive-local alignment parameters (Langmead and Salzberg 2012), with further analysis done using custom shell and R scripts. To facilitate comparisons among libraries, raw counts of mapped reads were normalized to the total number of reads passing both length (18–48 nt) and quality criteria (5 base sliding window with average quality above 20).

### Experimental design and statistical analysis

Experiments were conducted using completely randomized (*N. benthamiana*) or randomized complete block (*A. cruentus*) designs. Biological replication varied by experimental objective and is indicated in the figure legends. Generally, 2–6 biological replications were utilized. For gene expression and silenced-area measurements, each rep was sampled and assessed individually. For small RNA sequencing, the biological replications were pooled by treatment. All data analysis and graphical displays were completed using JMP12 (SAS, Cary, NC, USA). Data are displayed as means ± standard error of the mean and were analyzed using ANOVA. Means were separated using Student’s *t* test (*α* = 0.05) or contrasts as indicated in the figured legends.

#### *Author contribution statements*

ERH, CH, BH, MJ Bauer, WZ and JD conceived the research approaches; BH, WZ, MJ Bauer, ERH, JTM, PHH, RS and DNT performed research; ERH, CH, BH, WZ, AC-Y and JD supervised the experiments; ABI, JO and MJ Bennett developed the RNA delivery method; BDE analyzed sRNA data; JD wrote the article with contributions from BH, WZ, AC-Y, DNT and BDE; JD agrees to serve as the author responsible for contact.

## Supplementary Information

Below is the link to the electronic supplementary material.

Supplementary file1 (DOCX 23 kb)

## Data Availability

The datasets generated during and/or analyzed during the current study are available from the corresponding author on reasonable request.
