# Supplementary material for: Topically delivered 22 nt siRNAs enhance RNAi silencing of endogenous genes in two species
Source: Planta. 2021 Aug 26;254(3):60. doi: 10.1007/s00425-021-03708-y (PMC8390415; doi:10.1007/s00425-021-03708-y)
Supplement: Supplementary file 1 — Supplementary file1 (DOCX 23 kb) [file 425_2021_3708_MOESM1_ESM.docx]

**Supplementary Tables**

**Table S1.** Accession numbers for genes used in this study.

| **Species** | **Name** | **Sequence ID** | **Database URL** |
| --- | --- | --- | --- |
| Aequorea victoria | *GFP* | KY464890.1 | https://www.ncbi.nlm.nih.gov/nucleotide/ |
| *Nicotiana benthamiana* | *CHL-I* | Nbv5.1tr6204879 | http://benthgenome.qut.edu.au/ |
| *Nicotiana benthamiana* | *GUN4* | Nbv5.1tr6225868 | http://benthgenome.qut.edu.au/ |
| *Nicotiana benthamiana* | *CHL-H* | Nbv5.1tr6207395 | http://benthgenome.qut.edu.au/ |
| *Amaranthus hypochondriacus* | *CHL-H* | AHYPO_005471-RA | https://phytozome.jgi.doe.gov/pz/portal.html |

**Table S2.** *GFP* 5’ probe sequence for RNA gel blot.

CACTGGAGTTGTCCCAATTCTTGTTGAATTAGATGGTGATGTTAATGGGYACAAATTTTCTGTCAGTGGAGAGGGTGAAGGTGATGCAACATACGGAAAACTTACCCTTAAATTTATTTGCACTACTGGAAAACTACCTGTTCCATGGCCAACACTTGTCACTACTTTCTCTTATGGTGTTCAATGCTTTTCAAGATACCCAGATCATATGAAGCGGCACGACTTCTTCAAGAGCGCCATGCCTGAGGGATACGTGCAGGAGAGGACCATCTTCTTCAAGGACGACGGGAACTACAAGAC

**Table S3.** RT-qPCR primer and probe sequences for transcript measurements (Sequences are 5’-3’). MGB, Minor groove binding probe.

| **GENE ID** | **FWD PRIMER** | **REV PRIMER** | **PROBE** |
| --- | --- | --- | --- |
| *EF1a* | CACGCATTGCTTGCTTTCA | TCCATCTTGTTACAGCAGCAAATC | CTTGGTGTCAAGCAAA (MGB) |
| *UKN1* | ACTTGCTGAAGGTCTCTGTTG | AAGAGCTTTCCACCGATTCTC | TCTTTGGCCTTCCCCTACATTGATGC |
| *CHL-H* | CCAGGGCATTTTACGAGATGTG | TGCTGATATTGCTCCACGTGATG | SYBR Detection |
| *CHL-I* | AGCGTTGGCTGCACTAAAAGG | TTGGGAATGACAGTGGCGATAT | SYBR Detection |
| *GUN4* | GAGTAGCATTTTCACTCATCCTGCTT | CTTCCCCAGAAATACTTCCTTGTTTC | AGTTGAAGAAGGAGAAGAA (MGB) |
